# ZNF750 exerted its Antitumor Action in Oral Squamous Cell Carcinoma by regulating E2F2

**DOI:** 10.7150/jca.63919

**Published:** 2021-10-25

**Authors:** Hong-Li Yang, Cong Xu, Yi-Kun Yang, Wen-Qiang Tang, Min Hong, Li Pan, Hai-Ying Chen

**Affiliations:** 1 Central laboratory of Liaocheng People's Hospital, Liaocheng, 252000, P.R. China.; 2 School of Chemistry and Chemical Engineering, Liaocheng University, Liaocheng 252059, P. R. China.

**Keywords:** Oral squamous cell carcinoma (OSCC), Zinc-finger protein 750, E2F transcription factor 2 (E2F2), Enhancer of zeste 2 (Ezh2), PHD Finger protein 19 (PHF19)

## Abstract

Cell cycle activator E2F transcription factor 2 (E2F2) play a key role in tumor development and metastasis. Previous RNA sequence analysis (GSE134835) revealed E2F2 was significantly reduced by Zinc-finger protein 750 (ZNF750) in oral squamous cell carcinoma (OSCC). This study was aimed to determine the involvement of E2F2 in antitumor action of ZNF750. The nude mouse xenograft model was established by subcutaneously injection of stable cell line CAL-27^oeZNF750^ or CAL-27^shZNF750^. Xenograft tumor volume and tumor weight was measured. The expression of E2F2, transcriptional repressors such as enhancer of zeste 2 (Ezh2), PHD finger protein 19 (PHF19), and the genes related to cell proliferation or metastasis was studied *in vivo* or *in vitro*. Luciferase assay was performed to investigate regulation effect of ZNF750 on E2F2 luciferase activity. The involvement of E2F2 in the antitumor action of ZNF750 was studied by cotransduced ZNF750 with E2F2 lentivirus. The tumor growth and metastasis was repressed by ZNF750 manifested by reduced tumor size, tumor weight and the genes related to cell proliferation and metastasis. However, all of these were reversed by knockdown of the ZNF750 gene. Furthermore, E2F2 luciferase activity was inhibited by ZNF750. E2F2 partly blocked the antitumor action of ZNF750 manifested by increased self-renewal, invasion, migration, elevated Ezh2 and MMP13 protein expression in ZNF750 + E2F2 groups. However, silenced E2F2 further enhanced the antitumor action of ZNF750. ZNF750 depressed E2F2 activity and played a critical role in regulating transcriptional repressors for inhibiting the OSCC growth and metastasis in OSCC.

## Introduction

Oral squamous cell carcinoma (OSCC) is one of the most common malignant tumors worldwide, and approximately 90% of the total oral malignancies are squamous cell carcinomas 1. OSCC poses significant mortality and morbidity in the patients 2, therefore, it is urgently to develop new targeted therapy strategies.

Transcription factor Zinc-finger protein 750 (ZNF750) is a remarkable lineage-specific tumor suppressor gene shown to have a relationship with squamous cell carcinoma 3. Previous study had testified that ZNF750 was reduced or deleted in OSCC tissues, as a potential tumor suppressor, ZNF750 inhibited cell proliferation and cell metastasis in CAL-27 cells 4, 5. RNA sequence (NCBI/GEO/GSE134835) analysis showed that PHD finger protein 19 (PHF19), enhancer of zeste 2 (Ezh2) and E2F transcription factor 2 (E2F2) was downregulated by ZNF750 4.

Deregulations of PHD finger protein 19 (PHF19) and enhancer of zeste 2 (Ezh2) implied in epigenetic regulation have been frequently associated with cancers 6. PHF19, also known as PCL3 (polycomb-like 3), is a member of the polycomb-like (PCL) family with PHD finger domain. The increased PHF19 expression correlates with advanced cancers 7. PHF19 is polycomb repressive complexes 2 (PRC2) associated factor, which form sub-complexes with PRC2 three essential core components, including suppressor of zeste 12 homolog (SUZ12), embryonic ectoderm development (EED), and enhancer of zeste 1/2 (Ezh1/2), to modulate the activity of PRC2 8.

PRC2 is one of the multimeric polycomb group (PcG) protein complex. PcG proteins are essential epigenetic repressors. They form two major protein complexes, called polycomb repressive complexes 1 and 2 (PRC1 and PRC2) whose function is to maintain transcriptional repression 9. PRC2 is mainly trimethylate lysine 27 of histone H3 (H3K27me3). Trimethylation of H3K27 correlates with transcriptionally repressed chromatin. Ezh2 functions as a H3K27 methyltransferase when comprising the PRC2 10. Over-expression of Ezh2 has been reported in many cancers. It was testified that Ezh2 promoted cell proliferation, migration, invasion and metastasis of cancer cells 11.

Ezh2 are E2F transcription factors-regulated genes 12. E2F is a family of transcription factor proteins that have been implicated in multiple biological functions in human cancer 13. E2F are divided into two subfamilies: E2F transcription factors are divided into two subfamilies: transcription activators (E2F1, E2F2, E2F3a) and repressors (E2F3b, E2F4, E2F5, E2F6, E2F7 and E2F8). E2F2 is a member of E2F family of transcription activators 14. High E2F2 expression was correlated to worsen overall survival 15. Our previous RNA sequence analysis has found that ZNF750 has the ability to reduce E2F2 expression 4.

Currently, the transcriptional regulation of ZNF750 in OSCC is still unclear, and it remains to be fully determined the antitumor action of ZNF750 was relevance to its regulation on E2F2. The present study was to explore the mechanism of ZNF750 for inhibiting the cell proliferation or metastasis in OSCC cell line CAL-27 cells and xenograft model in nude mice.

## Materials and methods

### Animals, cell lines and plasmids

The five-weeks-old male BALB/c-nu mice weighing 16-17 g (certificate number SCXK Beijing 20160006) were obtained from the Beijing Vital River Laboratory Animal Technology Co., Ltd (Beijing, China). The mice were kept under conventional housing conditions (22 ± 1 °C, 40-70% humidity) with food and water supply* ad libitum* and 12 h day/dark cycle. The mice were acclimated for one week before the beginning of the experiment. The experimental protocol was approved by Liaocheng People's Hospital Research Ethics Committee. All studies with animals were treated in accordance with the “Guide for the Care and Use of Laboratory Animals”.

The 293T cells and oral squamous cell carcinoma (OSCC) cell line CAL-27 cells were purchased from Shanghai Zhong Qiao Xin Zhou Biotechnology Co., Ltd (Shanghai, China) and Procell Life Science & Technology Co., Ltd (Wuhan, China) respectively. The lentiviral packaging plasmids psPax2, VSV-G and pRSV-Rev were given as a kind gift by Dr Padraig Strappe (Central Queensland University, Australian). The plvx-PGK-Puro lentiviral vector backbone (oe-Con), plvx-ZNF750-PGK-Puro (oe-ZNF750) and plvx-E2F2-PGK-Puro (oe-E2F2) was purchased from Biowit Technologies (Shenzhen, China). The hU6-MCS-CMV-Puromycin-negative control (sh-Con), hU6-MCS-CMV-Puromycin-shZNF750 (sh-ZNF750) silenced the ZNF750 gene, hU6-MCS-CMV-Puromycin-shE2F2 (sh-E2F2) silenced the E2F2 gene and the renilla reniformis plasmids were purchased from Shanghai GeneChem Co., LTD. (Shanghai, China). The plasmids pcDNA3.1A-ZNF750 and PGL3-E2F2-promoter (-2500 to +100) for luciferase assay was purchased from Sangon Biotech Co., Ltd. (Shanghai, China).

### Cell cultures and treatment

The 293T cells and CAL-27 cells were grown in DMEM media supplemented with 10% FBS, streptomycin (100 mg/ml) and penicillin (100 IU/ml) at 37 °C in a humidified incubator with 5% CO_2_. All experiments were performed with mycoplasma-free cells. The CAL-27 cells growing at an exponential phase were randomly divided into four groups: Control (oe-Con and sh-Con) groups were transduced with oe-Con or sh-Con lentivirus respectively. Oe-ZNF750 (over-expression ZNF750 gene) and sh-ZNF750 (knockdown of the ZNF750 gene) groups were transduced with oe-ZNF750 or sh-ZNF750 lentivirus respectivly. For investigating the antitumor action of ZNF750 in CAL-27 cells was involved by E2F2, additional group Z+E (ZNF750+E2F2, over-expression of ZNF750 and E2F2 gene), Z+shE (over-expression of ZNF750 and silenced E2F2 gene) and oe-sh-Con (co-transduced with oe-Con and sh-Con lentivirus) was included.

### Lentiviral packaging and cell infection

The lentiviral packaging and infection was performed as we described previously 16. Briefly, lentiviral particles were produced in 293T cells by transfection with Lipofectamine 2000 (Thermo Fisher Scientific). Lipofectamine 2000/DNA complexes were added into 293T cells with the addition of caffeine (final concentration of 4 mM) to achieve higher titer lentivirus 17. Lentivirus-containing supernatant was collected at 48 and 72 h post-transfection, filtered and concentrated using SBI's one-step virus concentration solution, PEG-it TM (SBI, USA). The CAL-27 cells grown at 30-50% confluence were infected with the oe-Con, oe-ZNF750, sh-Con, sh-ZNF750 lentivirus respectively, or cotransduced oe-ZNF750 with oe-E2F2 or sh-E2F2, oe-Con with sh-Con respectively in the presence of Polybrene (5 μg/ml, Sigma). Cells were allowed to recover for 48 h before being subjected to puromycin selection to obtain a stable cell line CAL-27^oeZNF750^, CAL-27^shZNF750^, CAL-27^oeZNF750+oeE2F2^, and CAL-27^oeZNF750+shE2F2^. The stable cell line CAL-27^oeCon^, CAL-27^shCon^ and CAL-27^oe-sh-Con^ using as a control.

### Animal models and tumor xenograft growth

To evaluate the antitumor effect of ZNF750 in the nude mice, 20 nude mice were randomly divided into four groups (n=5 of each), oe-Con, oe-ZNF750, sh-Con and sh-ZNF750 groups. The stable cell line CAL-27^oeCon^ and CAL-27^oeZNF750^, CAL-27^shCon^ and CAL-27^shZNF750^ cells (3×10^6^) were inoculated right armpit of nude mice to construct xenografts tumor model. The needle was stopped internally for 5 sec, rotated and pulled out to avoid leakage of the cell suspension. The activity, diet and mental state of nude mice were observed daily. The tumor volume was measured by a caliper every 6-7 days and calculated using the formla length × width^ 2^ × 1/2. At the end of the experiment, the mice were euthanized 40 days later following inoculation. Tumors were excised and weighed to evaluate tumor growth, and then were fixed or frozen under liquid nitrogen for quantitative real-time PCR (qPCR) and western blot analysis.

### Immunohistochemistry

The antigen identified by monoclonal antibody Ki67 and proliferating cell nuclear antigen (PCNA) protein expression in xenograft tumor samples was investigated by immunohistochemistry to evaluate the proliferation of cancer cells. Briefly, xenograft tumor samples were isolated from surrounding normal tissues. Fresh tissue samples were fixed in 10% formaldehyde and embedded in paraffin, Serial 5 µm sections were cut, deparaffinized in xylene, and hydrated through an ethanol series. Endogenous peroxidase was quenched in 3% hydrogen peroxide for 15 min incubation at room temperature. The sectioned slides were incubated with anti-Ki67 (1:16,000) and anti-PCNA (1:1000) antibody antibodies (all from Proteintech) at 4 °C overnight. Then the sections were incubated with KIT-5010 MaxVision ^TM^ HRP-Polymer anti-Mouse/Rabbit IHC Kit (Maxin-Bio, Co., Fuzhou, China) for 15 min. The slides were stained using a DAB Chromogen Substrate Kit (Maxin-Bio, Co., Fuzhou, China) for 3-5 min, and sections were counterstained with hematoxylin to identify nuclei. Images were acquired using a digital camera under the microscope (CKX71, Olympus).

### Quantitative real-time PCR (qPCR)

Extraction of total RNA was performed using TRIzol^®^ reagent (Thermo Fisher Scientific). RNA purity was detected by NanoDrop 2000 (Thermo Fisher, America). RNA (1 μg) was reverse-transcribed to cDNA using a PrimeScript® RT Kit in 20 μl reactions. The cDNA product was diluted 2-folds, aliquoted, and stored at -80 °C. QPCR was performed on an ABI 7500 Sequence Detection System (Applied Biosystems) using SYBR^®^ Premix Ex Taq™ II Kit (all from Takara, Dalian, China). Amplification parameters for qPCR was 30 sec pre-incubation at 95 °C for one cycle, followed by 40 cycles of 95 °C for 5 sec and 60 °C for 34 sec. Table 1 summarizes the sequences of primers (Sangon Biotech) used in this study. The fold changes of genes were normalized to the housekeeping gene GAPDH by the 2-^ΔΔCT^ method. Samples were analyzed in triplicate and each experiment was repeated for at least three times.

### Western blot

Total protein was extracted from culture cells or tumor tissue from xenograft model of OSCC using 100 μl of ice cold lysis buffer including PMSF. The cell lysates were centrifuge for 5 min at 12000 × g at 4 °C and supernatant was collected as total protein. Protein concentrations were determined using the BCA protein assay kit (all from Beyotime, Jiangsu, China). Equal amount of proteins (15 µg) was used for Western blot. The proteins were denatured in 1× SDS - PAGE sample buffer (Beyotime, Jiangsu, China) for 5 min at 95 °C. Then, the denatured protein was electrophoresed on a 10% sodium dodecyl sulfate-polyacrylamide electophoresis (SDS-PAGE) and was transferred to polyvinylidene difluoride membranes (Millipore, Bedford, MA) after electrophoresis. Nonspecific bindings to the membranes were blocked with 5% (w/v) skimmed milk in TBST (Tris-buffered saline-Tween 20) at room temperature for 1 hour, and then incubated with the appropriate primary antibodies, anti-ZNF750, anti-E2F2 (1:1000, all from Abcam), anti-PHF19 (1:1000), anti-Ezh2 (1:2000), anti-cyclin D1 (1:5000 dilution), anti-matrix metalloproteinase (MMP) 9 (1:500, all from Proteintech), and mouse monoclonal anti-β-actin antibody (1:10000, Proteintech) overnight at 4 °C. After washing with TBST for three times, the membranes were incubated with species specific horseradish peroxidase coupled secondary antibodies for one hour. After washing, the membranes were visualized by ECL western blot detection reagents (Beyotime, Jiangsu, China). Quantification of the protein bands was visualized and analyzed with AlphaView analysis system (ProteinSimle, USA). The β-actin antibody was used as protein loading control. The values of ZNF750, PHF19, Ezh2, E2F2, cyclin D1 and MMP9 proteins expression were normalized against β-actin.

### Luciferase assay

To investigate the regulation effect of ZNF750 on E2F2, the Dual Luciferase Reporter Assay Kit (E1910; Promega, Mannheim, Germany) was used to check luciferase and renilla signals according to a protocol provided by the manufacturer. Cells were divided into two groups: control groups (co-transfected with pcDNA3.1A, renilla reniformis plasmids and PGL3-E2F2-promoter) and ZNF750wt group (co-transfected with pcDNA3.1A-ZNF750, renilla reniformis plasmids and PGL3-E2F2-promoter). Luciferase activity of cell lysates were analyzed at 72 h after transfection using the GloMax^®^ Navigator (Promega), and the relative luciferase activity was determined as the quotient of firefly luciferase and renilla luciferase activity. The folds change of E2F2 luciferase activity was calculated against control groups. The analysis was performed in triplicate.

### Evaluation of E2F2 involvement in the antitumor action of ZNF750

For investigating the antitumor action of ZNF750 in CAL-27 cells were involved by E2F2, cell propagation, invasion, migration, Ezh2 and MMP13 protein expression were investigated by cell counting kit-8 (CCK-8), tumor sphere, colony formation assay, transwell assay, western blot and flow cytometry assay respectively. The CAL-27 cells were divided into seven groups: oe-Con, oe-ZNF750, sh-Con, sh-ZNF750, Z+E (co-transduced with ZNF750 and E2F2 virus), oe-sh-Con, and Z+shE (co-transduced with ZNF750 and sh-E2F2 virus) groups.

To evaluate the tumor propagation potency and self-renewal of cells, cells viability, tumor sphere and colony forming assays was performed. For cells viability, it was evaluated by CCK-8 assay (Beyotime, Jiangsu, China) according to the manufacturer's instructions. Briefly, 10 μl of CCK-8 solution was added to each well during the 2 h of culture at 37 °C, and the absorbance in each well was measured at 450 nm using a 96-well Multiskan MK3 microplate reader and experiments were repeated three times.

For tumor sphere formation, each group of cells (1 cells/μl) was trypsinized, counted, and cultured in ultra-low adherent six well plate (Corning Costar) with serum free DMEM medium supplemented with B27 serum free supplements (1:50; Invitrogen, Thermo Fisher Scientific, Inc.), 20 ng/ml human recombinant epidermal growth factor and 20 ng/ml basic fibroblast growth factor (bFGF) (all from PeproTech, Inc., Rocky Hill, NJ, USA). The spheroid formation was imaged and counted after 7 days of culture.

For colony formation, the cells were seeded in six well plates at low density (500 cells/well) in triplicate, and cultured for 7 days. The plates were then washed with PBS and fixed with 4% paraformaldehyde for 30 min followed by staining with 0.5% crystal violet for 1 min. after washed with PBS, and the images of each well were captured and counted by AlphaView (ProteinSimple, Santa Clara, CA, USA). The test was repeated for three times.

The cell invasion and migration assay was performed as we described previous 5, 16. For cell invasion assay, Corning transwell chambers with polycarbonate membrane (8 μm pore size) were used to evaluate the cell invasion, and matrigel (BD Biosciences, Franklin Lakes, NJ, USA) was used as the substrate for invasion. Cells that invaded to the lower surfaces of the membrane were fixed with 4% formaldehyde, stained with 0.1% crystal, and visualized under light microscopy (CKX71, Olympus). The average number of invasion cells per field was assessed by Image-Pro Plus 6 software. Cell migration assay use a similar approach without matrigel coating. Three independent experiments were performed.

The protein expression of ZNF750, E2F2 and Ezh2 was investigated by western blot as we described above. MMP13 positive cells were assayed by flow cytometry. Each group of stable cell line cells (1×10^6^) was washed twice with ice cold PBS, resuspended and incubated cells in BD Cytofix/Cytoperm™ solution at a concentration of 1×10^6^ cells/0.5 ml for 20 min on ice. After washed twice with BD Perm/Wash™ buffer (1×), the cells were stained with 5 μl primary rabbit anti-MMP13 (Abcam) at 37 °C for 20 min, and then incubated with 5 μl secondary antibody Alexa 488 goat anti-rabbit IgG (H+L) for 20 min. Samples were analyzed by the BD FACSDiva software.

### Statistical analysis

The values were expressed as means ± SD. Data were analyzed by one-way ANOVA, followed by a post hoc SNK-*q* test using the SPSS 23.0 statistical package. When comparing two conditions, the data were analyzed by Student's t-test between two groups. *P* < 0.05 was considered a significant difference.

## Results

### E2F2 and transcriptional repressors were repressed by ZNF750 *in vivo* and *in vitro*

**Figure 1:**
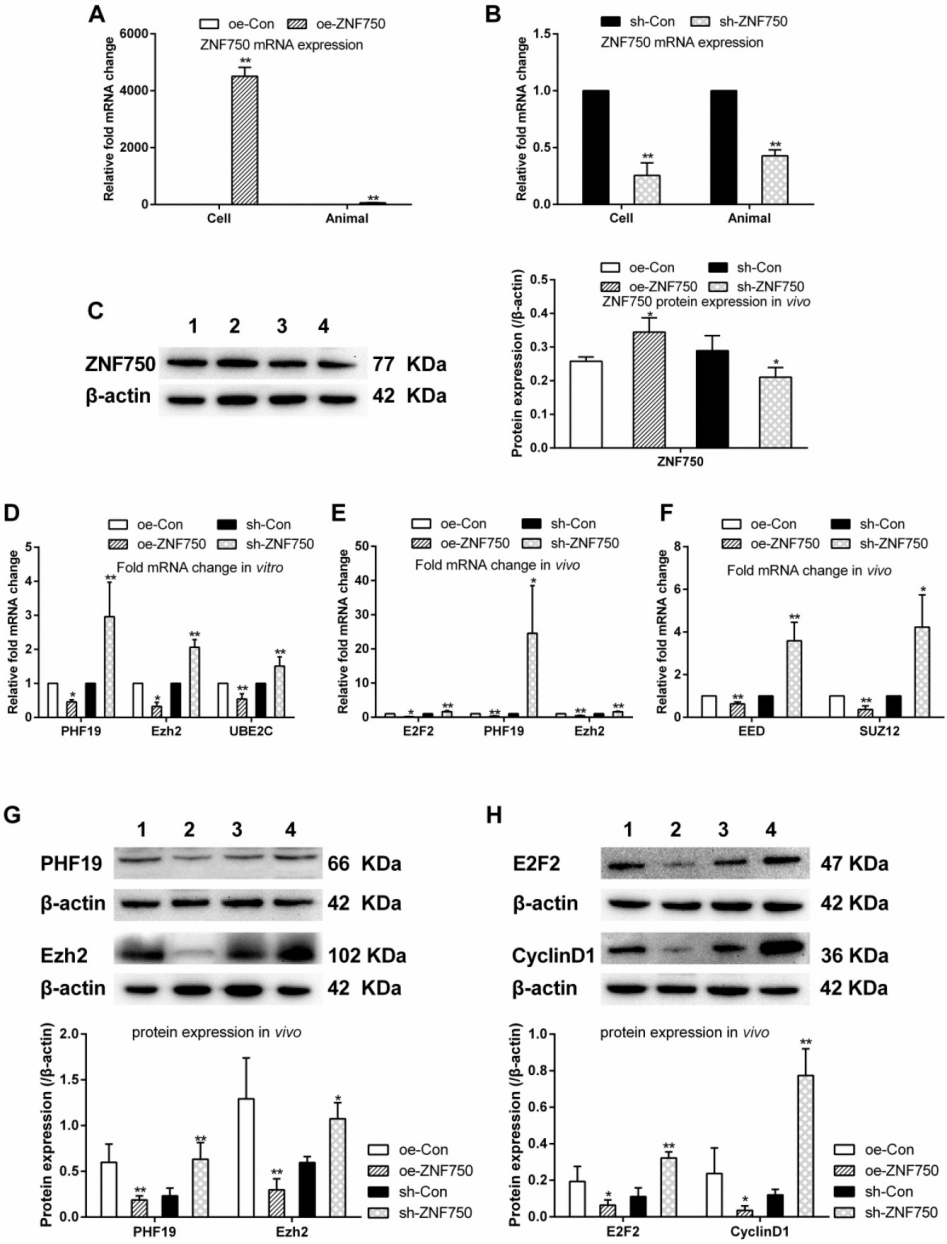
** ZNF750 inhibited the transcriptional repressors and cell cycle related genes *in vivo* and *in vitro*. A-C:** ZNF750 mRNA and protein expression in CAL-27 cells or xenograft tumor samples from nude mice. D-F: The PHF19, Ezh2, UBE2C, E2F2, EED and SUZ12 mRNA expression in CAL-27 cells or in xenograft tumor samples. G-H: Effect of ZNF750 on transcriptional repressors (PHF19, Ezh2) and cell cycle activator (E2F2, cyclin D1) protein expression. * *p<*0.05, ** *p<*0.01. 1: oe-control groups, 2: oe-ZNF750 groups, 3: sh-control groups, 4: sh-ZNF750 groups. The experiment was repeated at least three times. E2F2, E2F transcription factor 2; EED, Embryonic ectoderm development; Ezh2, Enhancer of zeste 2; PHF19, PHD Finger protein 19; SUZ12, Suppressor of zeste 12 homolog; UBE2C, ubiquitin-conjugating enzyme E2C; ZNF750, Zinc finger protein 750.

In this study, the expression of ZNF750 was detected by qPCR and western blot *in vivo* or *in vitro* to confirm stable cell lines were obtained. The ZNF750 mRNA expression was significantly increased 4505.9-folds and 55.8-folds in the CAL-27^oeZNF750^ cells and xenograft tumor samples respectively, while knockdown of the ZNF750 gene leaded to 77.8% and 60% down-regulation of ZNF750 in the CAL-27^shZNF750^ cells and xenografts tumor samples. Furthermore, the protein expression of ZNF750 was increased in oe-ZNF750 groups and decreased in sh-ZNF750 groups respectively *in vivo* (Figure 1A-C). The present study showed that the transcriptional repressors (PHF19, Ezh2, EED, SUZ12) and UBE2C mRNA or protein expression was all downregulated by ZNF750, whereas, it was all upregulated in sh-ZNF750 groups *in vivo* or *in vitro* (Figure 1D-G). Moreover, the cell cycle activator E2F2 and cell cycle regulator cyclinD1 expression was repressed in oe-ZNF750 groups but it was increased in sh-ZNF750 groups compared to their matched control groups (Figure 1E, 1H).

### ZNF750 inhibited the cell growth *in vivo*

**Figure 2:**
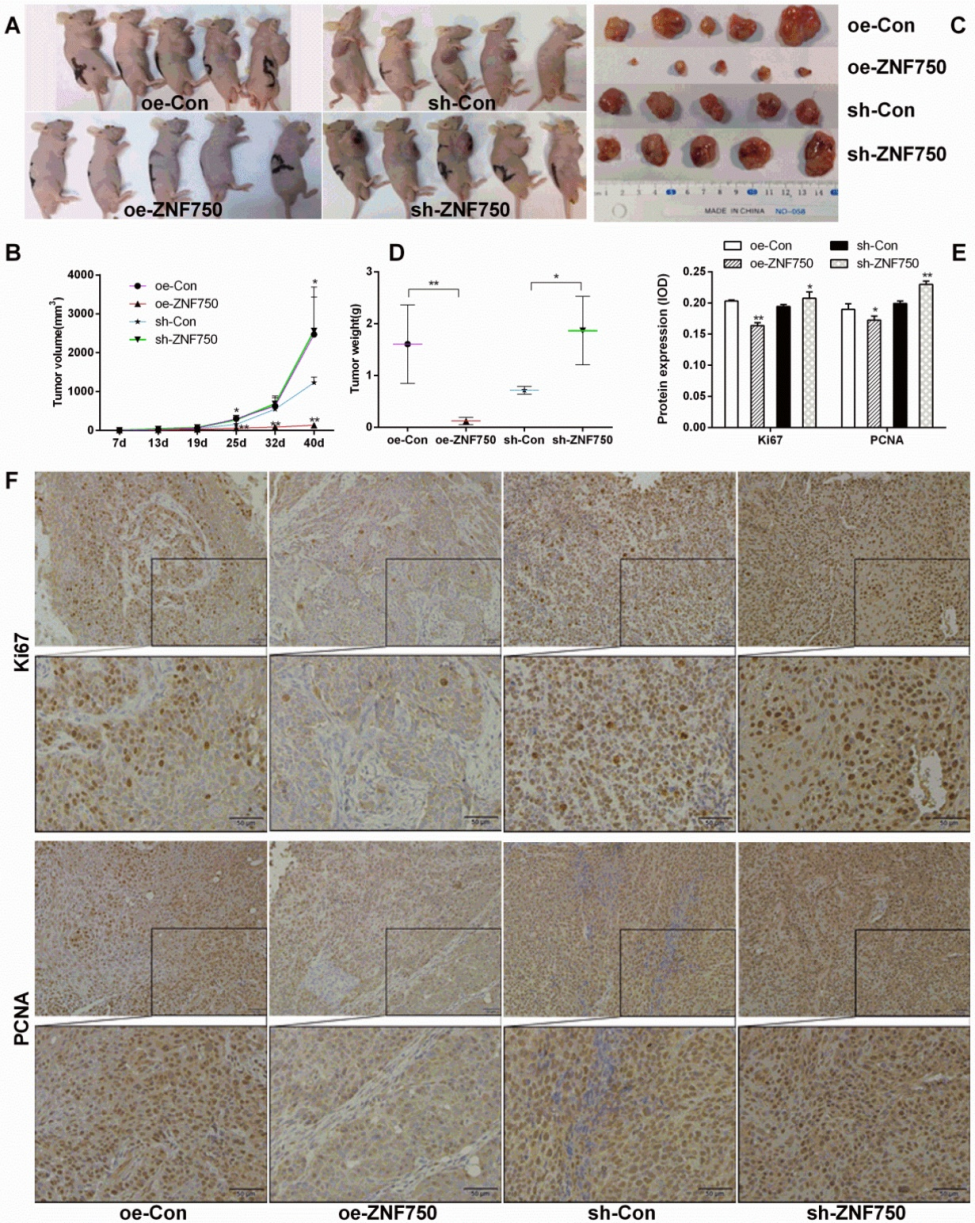
** Effect of ZNF750 on tumor volume and tumor weight. A-B:** The changes of tumor volume in each group (n=5 of each). The mice were euthanized 40 days later following inoculation. **C-D:** The tumor weight was reduced in oe-ZNF750 groups but elevated in sh-ZNF750 groups. **E-F:** The proliferation marker Ki67 and PCNA (Proliferating cell nuclear antigen) protein expression in xenograft tumor samples. Bar=20×. * *p<*0.05, ** *p<*0.01.

To confirm the inhibitory effect of ZNF750 on tumor growth *in vivo*, we subcutaneously injected the stable cell lines CAL-27^oeCon^, CAL-27^oeZNF750^, CAL-27^shCon^ and CAL-27^shZNF750^ into nude mice, and then the tumor volume was measured about weekly for 40 days. The tumor formation rate in the nude mice was 100% (20/20). The present study showed that the tumor volumes were smaller in oe-ZNF750 groups than in oe-con groups at check point, but it was bigger in sh-ZNF750 groups than in sh-Con groups (Figure 2A, 2B). Furthermore, the tumor weight was reduced in oe-ZNF750 groups but it was increased in sh-ZNF750 groups than in their matched control groups, which indicated that ZNF750 had significant inhibitory effect on tumor growth (Figure 2C, 2D). Moreover, the proliferation markers Ki67 and PCNA protein expression in xenograft tumor samples was lower in the oe-ZNF750 groups than in oe-Con groups, whereas, it was higher expressed in sh-ZNF750 groups than in sh-Con groups (Figure 2E, 2F). The expression level of Ki67 and PCNA in oe-ZNF750 and sh-ZNF750 groups was in consistent with the observation results from the tumor growth.

### ZNF750 repressed the genes related to metastasis *in vivo*

**Figure 3:**
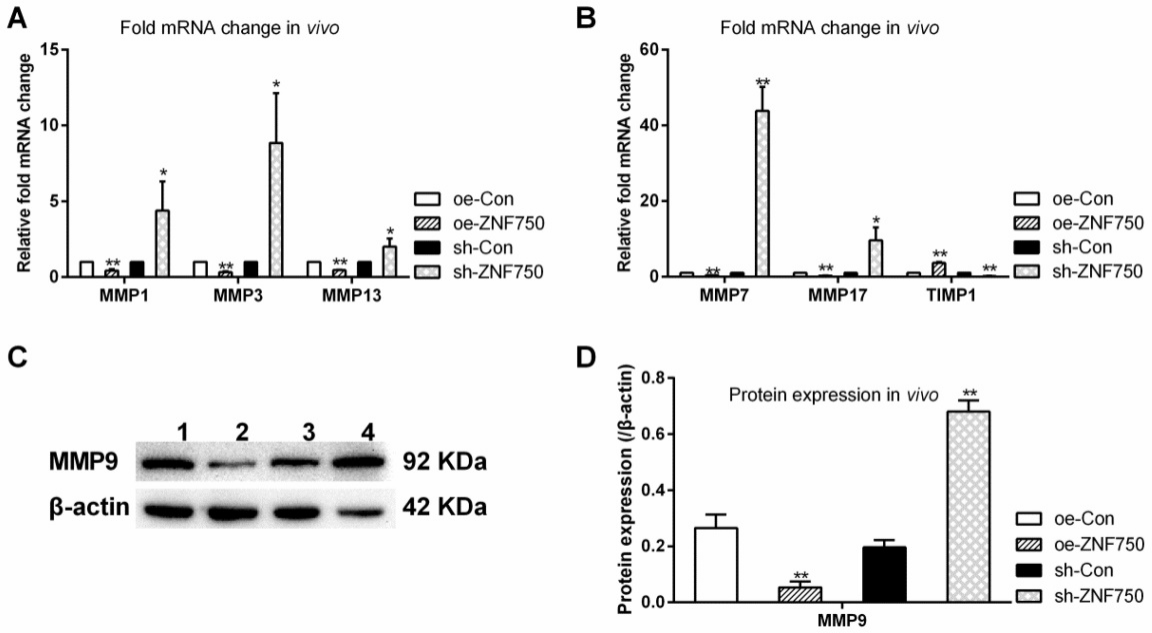
** Effect of ZNF750 on metastasis related genes expression *in vivo*. A-B:** The mRNA expression of MMPs (MMP1, MMP3, MMP7, MMP13, MMP17) and their endogenous inhibitor TIMP1 (Tissue inhibitor of metalloproteinase-1)*.* **C-D:** The protein expression of MMP9*.* * *p<*0.05, ** *p<*0.01. 1: oe-control groups, 2: oe-ZNF750 groups, 3: sh-control groups, 4: sh-ZNF750 groups. MMP, Matrix metalloproteinase.

The expression of metastasis related genes MMP1, MMP3, MMP7, MMP9, MMP13, MMP17 and their endogenous inhibitor tissue inhibitor of metalloproteinase-1 (TIMP1) was studied to evaluate the inhibitory effect of ZNF750 on cell metastasis *in vivo*. The results showed that the above mentioned matrix metalloproteinases (MMPs) expression were all reduced by ZNF750, whereas, it was all increased in sh-ZNF750 groups compared to their matched control groups. However, the MMP inhibitor TIMP1 was downregulated in sh-ZNF750 groups but was upregulated in oe-ZNF750 groups (Figure 3A-D).

### ZNF750 inhibited E2F2 luciferase activity

**Figure 4:**
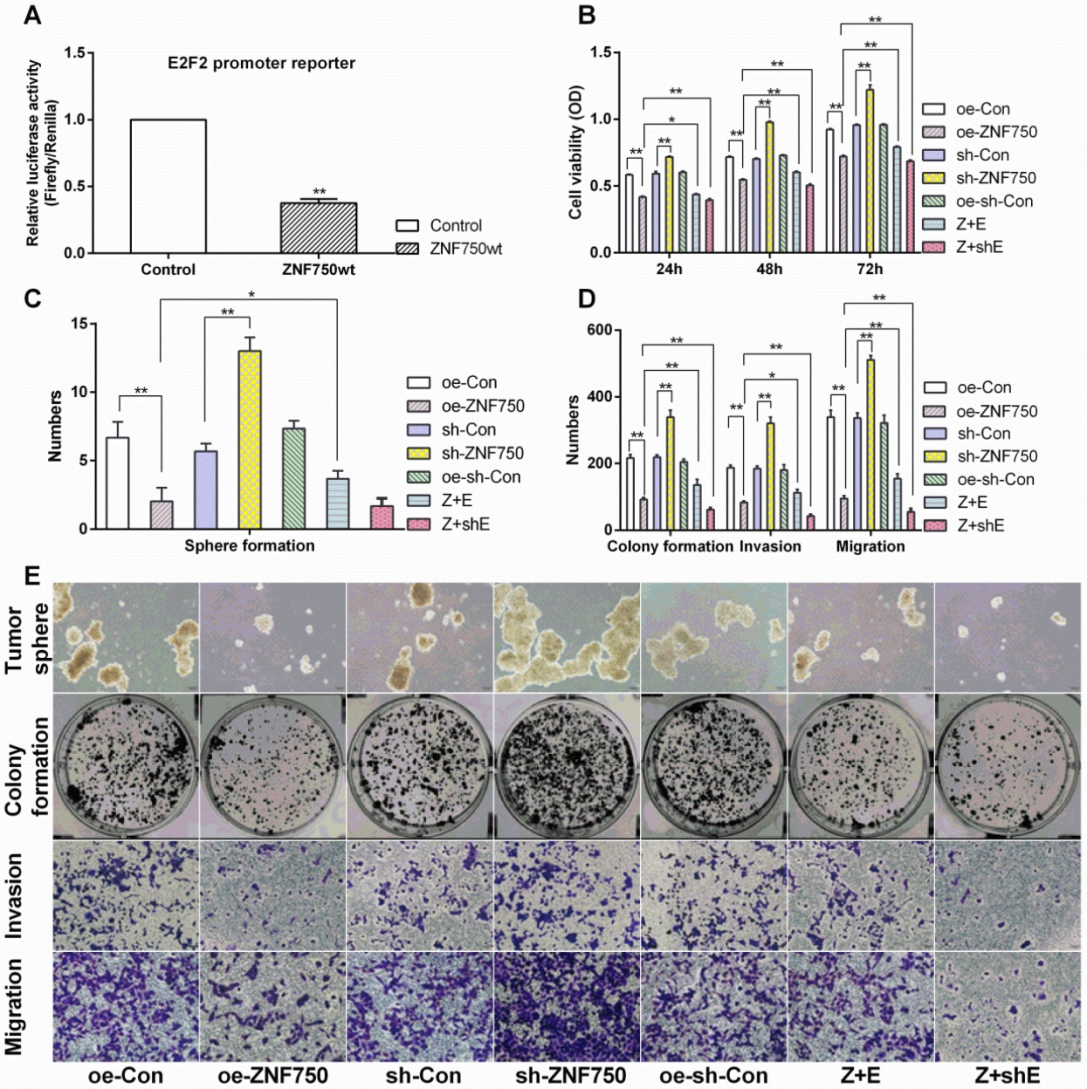
** The involvement of E2F2 on antitumor action of ZNF750. A:** ZNF750 negative regulated the E2F2 luciferase activity. Control groups: co-transfected with pcDNA3.1A, renilla reniformis and PGL3-E2F2-promoter plasmids, ZNF750wt groups: co-transfected with pcDNA3.1A-ZNF750, renilla reniformis and PGL3-E2F2-promoter plasmids. **B:** Cell viability, **C-E:** ZNF750 led to reduced self-renewal, cell invasion and migration in CAL-27 cells.

Luciferase reporter assay indicated that the E2F2 gene relative luciferase activity was significantly decreased in ZNF750wt groups compared to control groups (*p*<0.01), which indicated that ZNF750 negatively regulated the E2F2 expression (Figure 4A).

### E2F2 involved in the inhibitory action of ZNF750 on cell proliferation, invasion and migration

The present results indicated that ZNF750 could abolish the cell proliferation, invasion and migration ability, manifested by reduced cell viability (reduced about 1.40-folds), number of tumor sphere (3.34-folds), colony formation (2.33-folds), cell invasion (2.27-folds) and migration (3.58-folds) in oe-ZNF750 groups compared to oe-con groups, but it was all increased in sh-ZNF750 groups compared to sh-con groups. Furthermore, E2F2 partly blocked the antitumor function of ZNF750 manifested by increased cell proliferation, invasion and migration in Z+E groups compared to oe-ZNF750 groups. However, cotransduced sh-E2F2 with ZNF750 lentivirus further enhanced the antitumor function of ZNF750 in Z+shE groups (Figure 4B-E).

### E2F2 blocked the depressed expression of Ezh2 and MMP13 induced by ZNF750

**Figure 5:**
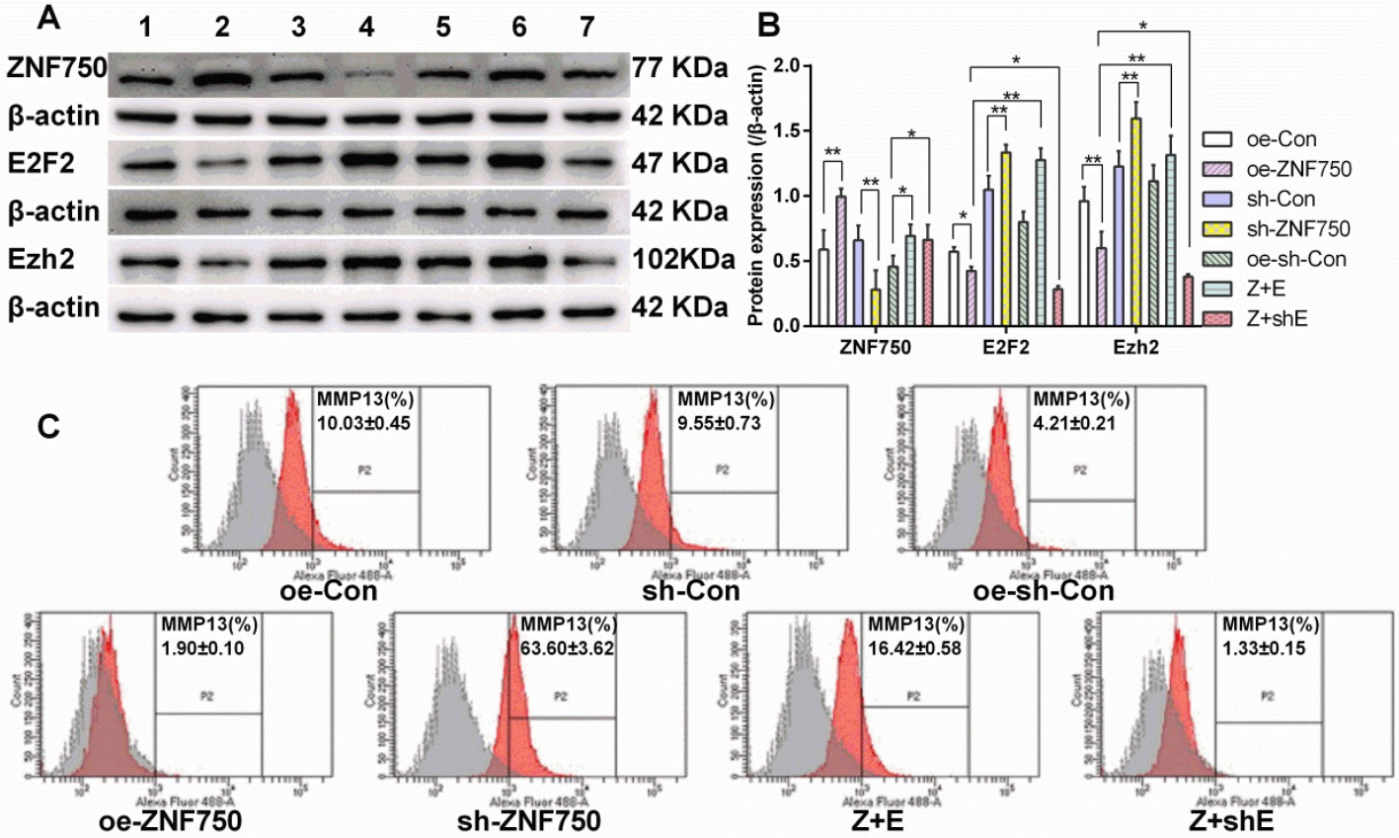
** E2F2 partly blocked the inhibitory function of ZNF750 on Ezh2 and MMP13 protein expression. A-B:** ZNF750, E2F2 and Ezh2 protein expression. **C:** MMP13 protein expression was detected by flow cytometry. ZNF750 reduced the MMP13 protein expression from 10.03% to 1.90%, however, E2F2 partly blocked the inhibitory function of ZNF750 on MMP13 expression (from 1.90% to 16.42%). * *p<*0.05, ** *p<*0.01. 1: oe-control groups, 2: oe-ZNF750 groups, 3: sh-control groups, 4: sh-ZNF750 groups. 5: oe-sh-control groups, 6: Z+E groups, 7: Z+shE groups. E2F2, E2F transcription factor 2; Ezh2, Enhancer of zeste 2; ZNF750, Zinc finger protein 750.

Western blot showed that E2F2 partly reversed the inhibitory effect of ZNF750 on transcriptional repressor Ezh2 expression. The Ezh2 protein expression was reduced in ZNF750 groups but it was elevated in sh-ZNF750 groups compared to their matched groups (Figure 5A, 5B). Moreover, compared to ZNF750 groups, Ezh2 protein expression were elevated in Z+E groups but slightly reduced in Z+shE groups (Figure 5A, 5B). The flow cytometry analysis further testified that MMP13 protein expression in CAL-27 cells was repressed (from 10.03% to 1.90%) by overexpression of ZNF750 but it was elevated (from 9.55% to 63.60%) by knockdown of the ZNF750 gene compared to their matched groups, and more importantly, cotransduced E2F2 with ZNF750 lentivirus partly blocked the inhibitory effect of ZNF750 on MMP13 protein expression (from 1.90% to 16.42%) compared to oe-ZNF750 groups (Figure 5C).

## Discussion

Our previous study had found that the potential tumor suppressor ZNF750 inhibited cell cycle activator E2F2 and transcriptional repressors (PHF19, Ezh2) expression analyzed by RNA sequence (NCBI/GEO/GSE134835) 4. In this study, we elucidated the antitumor mechanism of ZNF750 on OSCC *in vivo* or *in vitro*. The present study revealed that knockdown of the ZNF750 gene enhanced the expression of E2F2, transcriptional repressors and UBE2C in CAL-27 cells or xenograft model in nude mice. On the contrary, increased ZNF750 expression leaded to decreased expression of it *in vivo* or *in vitro*. These observations are consistent with our previous RNA sequence analysis (NCBI/GEO/GSE134835) that transcriptional repressor PHF19 and Ezh2 expression was repressed by ZNF750 4.

PHF19 (PHD finger protein 19) is PRC2 associated factors that form sub-complexes with PRC2 core components to modulate the enzymatic activity of PRC2 8. PRC2 is the major H3K27 methyltransferase and is responsible for maintaining repressed gene expression patterns throughout development 18. Thus, PHF19 functions as a transcriptional repressor, and play an important role in regulating transcription and histone demethylation 19. Decreasing level of PHF19 resulted in reduced H3K27me3 while overexpressing PHF19 led to increased H3K27me3 level 20. The PRC2 complex is composed of a trimeric core of Ezh1/2, EED and SUZ12, catalyzes the trimethylation of histone H3 at lysine 27 (H3K27me3) 21. PHF19 could form the PRC2 with Ezh2, EED, and SUZ12, knockdown of the PHF19 gene suppressed Ezh2 phosphorylation and proliferation in glioma cells 22. Therefore, the increased PHF19 with Ezh2, EED, and SUZ12 which form the PRC2 in sh-ZNF750 groups may result in abnormal repressed gene expression.

Ezh2 is downstream of the pRB-E2F pathway, amplified in cancer and strongly associated with tumor proliferation and aggressiveness 23, 24. Targeting Ezh2 markedly suppressed OSCC invasion 25. It was found that Ezh2 could interact with ubiquitin-conjugating enzyme E2C (UBE2C) 26, and UBE2C involved in head and neck tumorigenesis through cell cycle 27. Cell cycle inhibitor p21 caused growth arrest by inhibition of cyclin D1 28. Ezh2 suppressed the expression of p21 through histone methylation (H3K27me3) on the p21 promoter resulted in cell proliferation. E2F2 is a member of the E2F family of transcription factors. In addition to their well-characterized roles in cell cycle control, E2F2 play key roles in mediating tumor development and metastasis 29. Our previous study had revealed that cell cycle was involved in the antitumor effect of ZNF750 4. The current study were consistent with above studies manifested by overexpressed ZNF750 accompany with reduced E2F2, PHF19, Ezh2, EED, SUZ12, UBE2C and cyclinD1, and the vise verse for knockdown of the ZNF750 gene. Thus, the increased E2F2, transcriptional repressor PHF19, Ezh2, EED and SUZ12 in sh-ZNF750 groups could regulate the repressive transcriptional activity resulted in enhanced cyclinD1expression and cell growth.

In line with above studies, we proofed that the changes of ZNF750 expression led to the changes of tumor growth, which manifested by decreased tumor volume and tumor weight, reduced Ki67 and PCNA expression in oe-ZNF750 groups, but all of these was increased in sh-ZNF750 groups. It is well known that Ki67 and PCNA are proliferation markers. The elevated expression level of Ki67 and PCNA in oe-ZNF750 and diminished level in sh-ZNF750 groups was in consistent with the observation results from the tumor growth *in vivo*.

Metastasis has been identified as the main cause of high mortality in OSCC 30. MMPs and their endogenous inhibitors TIMPs regulate extracellular matrix degradation and synthesis critical for cancer cell metastasis. Imbalances of MMPs and TIMPs lead to progression of various diseases including cancer 31. Activated MMPs mediated matrix degradation and leading to tumor cell invasion 32. It has been reported that TIMP1 has strong inhibiting properties against MMP9 31. In the current study, a group of MMPs (including MMP1, MMP3, MMP7, MMP9, MMP13 and MMP17) were all decreased but TIMP1 was increased in ZNF750 groups. However, MMPs were elevated but TIMP1 was diminished in sh-ZNF750 groups. According to the above mentioned results, our observations proved the inhibitory effect of ZNF750 on cell metastasis.

The present study elucidated that E2F2 was downregulated by ZNF750 and involved in the antitumor action of ZNF750. Luciferase reporter assay indicated that ZNF750 regulated the E2F2 lead to reduced E2F2 relative luciferase activity, which testified the negative regulation effect of ZNF750 on E2F2. Overexpression of E2F2 partly blocked the inhibited effect of ZNF750 on cell proliferation, invasion, migration, Ezh2 and MMP13 protein expression. More recent work has found that knockdown of the ZNF750 gene significantly promoted cell proliferation, colony formation, migration and invasion in esophageal squamous cell carcinoma cells 33. Parallel in other cell type support a potential tumor suppressor role of ZNF750, the present study testified that ZNF750 attenuated cell invasion, migration and MMP13 positive population. Moreover, the cell viability, colony forming activity, and the self-renewal capacity of each group in CAL-27 cells was paralleled with the cancer cell proliferation in the nude mice. Furthermore, compared to ZNF750 groups, the cell proliferation, invasion and migration were augmented in Z+E groups. More importantly, E2F2 could block the inhibitory function of ZNF750 on transcriptional repressor, which manifested by increased Ezh2 protein expression in Z+E groups than in oe-ZNF750 groups, but the Ezh2 was further reduced in Z+shE groups. Therefore, we postulated that ZNF750 may be a crucial mediator on Ezh2, leading to reduced tumor growth, metastasis, and the E2F2 was involved in its regulation.

Taken together, the present study revealed a possible novel mechanism underlying malignant biological behavior caused by loss function of ZNF750 in OSCC* in vivo* or *in vitro*, and indicated that ZNF750 as a tumor suppressor plays a vital role in regulating transcriptional repressors and this function was related to depressed expression of E2F2 by ZNF750. The detail mechanism of ZNF750 on tumor suppressing in OSCC involved by E2F2 will be further investigated in the future.

## Figures and Tables

**Table 1:** The sequences of primers

Gene	Refseq Accession#	Direction	Sequence
E2F2	NM_004091.3	Forward	GGGCCCATCGAAGTCTACCT
E2F2		Reverse	ATGATGCTAGGGTCGGTGCT
EED	NM_003797	Forward	GTCCTGTGGTATGGATCATTCT
EED		Reverse	GTATCAAATCGCCTAACCATCG
Ezh2	NM_004456.4	Forward	AAATCAGAGTACATGCGACTGA
Ezh2		Reverse	GTATCCTTCGCTGTTTCCATTC
MMP1	NM_002421.4	Forward	TGGGCTGAAAGTGACTGGGA
MMP1		Reverse	ATGGCATGGTCCACATCTGC
MMP3	NM_002422.5	Forward	GGCCAGGGATTAATGGAGAT
MMP3		Reverse	TGAAAGAGACCCAGGGAGTG
MMP7	NM_002423.5	Forward	ACCGTGCTGTGTGCTGTGTG
MMP7		Reverse	AGTCCTGAGCCTGTTCCCACTG
MMP13	NM_002427.3	Forward	CACTTTATGCTTCCTGATGACG
MMP13		Reverse	TCTGGCGTTTTTGGATGTTTAG
MMP17	NM_016155.7	Forward	CACTCATGTACTACGCCCTCA
MMP17		Reverse	TGGAGAAGTCGATCTGGATGTC
PHF19	NM_015651.3	Forward	ACCCCAGTGACAGATCGAGG
PHF19		Reverse	AGAGGAGGCGCTGTTCTCAT
TIMP1	NM_003254.3	Forward	CATCACTACCTGCAGTTTTGTG
TIMP1		Reverse	TGGATAAACAGGGAAACACTGT
UBE2C	NM_007019.4	Forward	ACCCTCATGATGTCTGGCGA
UBE2C		Reverse	ATAGCAGGGCGTGAGGAACT
ZNF750	NM_024702.3	Forward	GCCACCATCTACTCGCCTTA
ZNF750		Reverse	GCAGGAAGTGTCTCGGGTCT
SUZ12	NM_015355	Forward	CAAACTGAAGCAAGAGATGACC
SUZ12		Reverse	GCTATGGCAGAGTTTAAGATGC

## Abbreviations

E2F2: E2F transcription factor 2
EED: Embryonic ectoderm development
Ezh2: Enhancer of zeste 2
H3K27me3: Trimethylate lysine 27 of histone H3
MMP: Matrix metalloproteinase
OSCC: Oral squamous cell carcinoma
PCL: Polycomb-like
PCNA: Proliferating cell nuclear antigen
PHF19: PHD Finger protein 19
PRC2: Polycomb repressive complexes 2
SUZ12: Suppressor of zeste 12 homolog
TIMP1: Tissue inhibitor of metalloproteinase-1
UBE2C: ubiquitin-conjugating enzyme E2C
ZNF750: Zinc finger protein 750
